# Designed and tailor-made double hydrophilic block copolymer-graphene nanoplatelet hybrids for reinforcing epoxy thermosets

**DOI:** 10.1038/s41598-024-59322-x

**Published:** 2024-04-16

**Authors:** Jitha S. Jayan, BDS Deeraj, Kuruvilla Joseph, Appukuttan Saritha

**Affiliations:** 1 Department of Chemistry, Amrita Vishwa Vidyapeetham, Amritapuri, Kollam, Kerala India; 2 Department of Chemistry, Indian Institute of Space Science and Technology Valiyamala, Thiruvananthapuram, Kerala India

**Keywords:** Nanocomposites, Polymer-matrix composites, Graphene, Fracture toughness, Rheological properties, Mechanical properties, Chemistry, Engineering, Materials science, Nanoscience and technology

## Abstract

Because of their propensity to build micellar nanostructures, amphiphilic block copolymers (ABCs) are an appropriate and unique toughening agent for epoxy systems individually on their own and in grafted form. The presence of epoxiphilic and phobic ends in ABCs is responsible for the self-assembly and the micellar structure. Nanofiller-grafted ABCs can effectively enhance the toughness of epoxy via the synergistic interaction of nanofillers and the ABCs. Even though there is sound literature supporting the effect of ABCs in epoxy, the action of double hydrophilic block copolymers (DHBC) in the epoxy matrix is less handled. Hence, the grafting of nanofillers in DHBCs and their subsequent role in tuning the properties of epoxy is a new concept. Hence this paper tries to bridge the gap via studying the effect of grafted fillers based on DHBCs in epoxy matrix. As a result, the current study focuses on the synthesis of double hydrophilic graphene nanoplatelets (rGO-g-DHBC) via nitrogen oxide-mediated polymerization for epoxy toughening application. The prepared rGO-g-DHBC was effectively utilized for epoxy toughening applications, resulting in a 457% improvement in toughness without compromising its inherent tensile strength. The mechanism behind the improved toughness was elucidated with the help of a scanning electron microscope, and the thermal, and rheological characteristics were studied.

## Introduction

Block copolymers and nanofillers are the most recent epoxy toughening agents^1–4^. ABCs are an excellent alternative for increasing the toughness of epoxy due to their behaviour as surfactants and their propensity to self-assemble into various nanostructures^5–8^. The enhanced surface area and aspect ratio of nanofillers are capable of imparting toughness to epoxy^9–13^. These two fillers can be employed to strengthen epoxy either in the grafted or bare form^14–17^. ABCs are widely used in epoxy due to their potential microphase separation and the consequent generation of nanostructures that can boost mechanical strength^18–21^. Although the block copolymers work well as toughening agents, they fall short of the required tensile strength in some situations where epoxy resin is used. Double hydrophilic block copolymers (DHBCs) are a new class of emerging block copolymers that have not yet been used for epoxy toughening^22–24^. These BCPs are characterized by the presence of two or more hydrophilic blocks, generally, one of the blocks is soluble in water and the other will be capable of interacting with another polymer^25^. By changing a few factors, including the pH, one of the blocks of DHBCs can be converted into a hydrophobic state, allowing them to self-assemble in water similar to ABCs.

Two water-soluble polymers were selected in the current work, polyethylene glycol (PEG) and polyvinyl pyrrolidone (PVP). DHBCs prepared using water-miscible blocks like PVP and PEG can be made to self-assemble in water by adjusting the pH. Thus it is possible to fashion the nanostructures despite the miscibility of the blocks of the DHBC with water. Consequently, it is referred to as amphiphilic block copolymers in several investigations^26,27^. The ability of this copolymer to self-assemble into a micelle in the water medium has been studied by various research groups whereas the miscibility of these double hydrophilic copolymers in an epoxy matrix has not yet been explored. From the previous study, it is clear that PVP and PEG homopolymers are miscible with epoxy resin^28–30^. Our earlier studies with these materials confirm the idea that both PVP and PEG-based homo polymer systems can enhance the overall performance of epoxy composites^31–34^.

Studies related to the grafting of amphiphilic block copolymers to Graphene Oxide (GO) to produce amphiphilic nanoplatelets have been reported^35–38^. As mentioned before, ABC grafted nanofillers have been used as effective toughening agents of epoxy^39^. However hydrophilic block copolymer graphene platelets have not yet been used for the toughening of epoxy which is the motivation behind this work. In the present study, we have focused on the synthesis of double hydrophilic graphene nanoplatelets by the *in-situ* polymerization of PVP-b-PEG block copolymers aiming at the toughening of epoxy. The miscibility in epoxy as well as its strong interaction with GO is quite an interesting property for the enhancement of the mechanical properties of epoxy. To the best of our knowledge, none of the works reported so far has used double hydrophilic graphene nanoplatelets for toughening the epoxy matrix. This is a maiden attempt to design, synthesize, and develop these hybrid material-based epoxy composites. This novel approach would unveil new dimensions in epoxy toughening.

## Materials and methods

### Materials

Epoxy resin with a Diglycidyl Ether of Bisphenol-A (DGEBA) base and the curing agent Diethylene Toluenediamine (DETDA) were procured from Aditya Birla's epoxy business (CAS No:68479-98-1). Sigma Aldrich provided 99% pure graphite powder, NVP monomer, ethylene glycol monomer, hydrogen peroxide (H_2_O_2_), sodium nitrate (NaNO_3_), hydrochloric acid (HCl), and potassium permanganate (KMnO_4_).

### Synthesis of double hydrophilic graphene nanoplatelets

**Figure 1:**
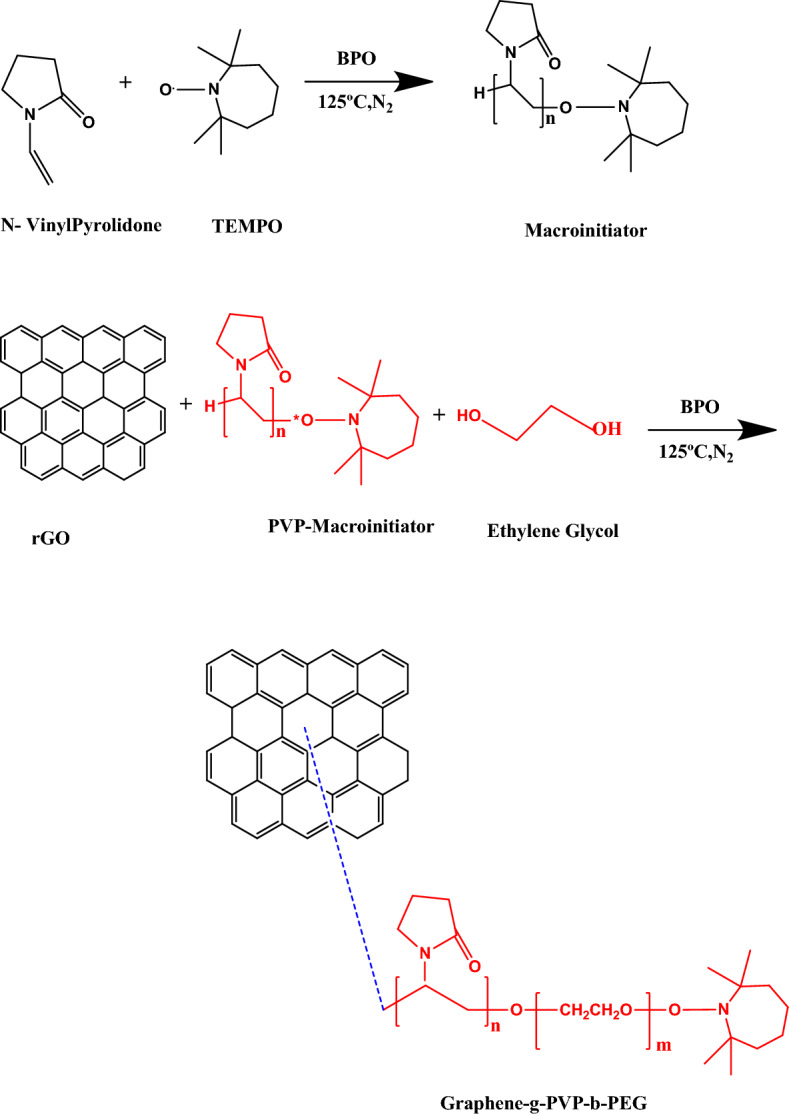
Schematic representation of the synthesis of rGO-g-DHBC block copolymer.

Graphene oxide is synthesized from graphite by following the well-known Hummer’s method as reported in our previous studies^40,41^. Reduced graphene oxide was prepared using the above synthesized GO as a precursor. GO is dispersed well in DMF employing sonication and then treated with NaBH_4_ at a temperature of 80 °C for four hours for the synthesis of reduced GO(rGO) sheets.The rGO thus obtained was washed several times with water and then dried. Hydrophilic nanoplatelets were synthesized by Reversible Addition Fragmentation Chain Transfer polymerization (RAFT). The detailed procedure is schematically represented in Fig. 1. In the first step, the PVP-macroinitiator is synthesized from N-vinyl pyrolidone (10 ml) by treating it with 0.285 g of 2,2,6,6-Tetramethylpiperidin-1-yl)oxyl(TEMPO) and 0.325 g benzoyl peroxide (BPO) at a temperature of 125 °C in N_2_ atmosphere. Following this, 0.175 g of PVP-macroinitiator, 2 g of reduced GO,1.12 g of ethylene and 0.35 g of BPO were dissolved in DMF and heated at a temperature of 125 °C in N_2_ atmosphere leading to the polymerization of ethylene glycol at one end of the PVP macroinitiator. The obtained black-colored mass was washed several times with DMF, and H_2_O and then oven dried.

### Synthesis of epoxy nanocomposites

Epoxy composites with GO/rGO-g-DHBC as fillers were prepared by dispersing the filler in acetone by sonication followed by the addition of DGEBA resin. After dispersing the filler well in the epoxy matrix by sonication, the solvent was removed using a vacuum desiccator. The curative DETDA (24.4Phr) was then mixed with the filler-loaded epoxy resin at a temperature of 80 °C, followed by pre-curing at a temperature of 140 °C for 2 h and post-curing at a temperature of 200 °C for 2 h.

### Spectroscopic characterizations and investigations

Nuclear magnetic resonance (NMR), Fourier transform infrared spectroscopy (FTIR), X-ray diffraction (XRD), X-ray photoelectron spectroscopy (XPS), and Raman spectroscopy were used to validate the production of rGO-g-DHBC. Perkin Elmer Spectrum 2 was utilized to carry out FTIR analysis in the range of 4000–500 cm^-1^ in ATR mode. Proton NMR studies were done using 800 MHz FT NMR instrument using dimethyl sulphoxide. With the aid of the Alpha 300 RA Raman equipment and a 532 nm laser source, the Raman spectrum of the produced materials was measured throughout ten accumulations ranging from 100 to 3000 cm^-1^ (DPSS-Nd:YAG). To compare the crystallinity of rGO-g-DHBC to GO, the 3 kW X'pertPRO equipment from PANalytical was used. Using radiation generated by Al K excitation, X-ray Photoelectron Spectroscopic (XPS) examination was performed using a DLD spectrometer ultra. SEM (Scanning Electron Microscope) and HRTEM (High-Resolution Transmission Electron Microscope) was used to examine the surface morphologies of cracked surfaces and rGO-g-DHBC (HRTEM). TESCAN VEGA3 SB was used to plot SEM images, while Jeol/JEM 2100 was used to plot HRTEM images with LaB6 as the source. Atomic Force Microscopy (AFM) pictures were obtained using WITec alpha 300RA, and non-contact mode analysis was employed during the process.

UTM (Instron 5984, Instron, USA) was used to test the elongation of break, tensile strength, and modulus of epoxy samples with dog bone shapes manufactured following ASTM standard D 638 ($165\times 12.7\times 3.2$ mm^3^) with a gauge length of 100 mm and a speed of 1 mm/min. The fracture toughness of the epoxy samples was evaluated using the same UTM device by following ASTM standard D 5045 at a crosshead speed of 10 mm/min. Single-edge notched specimens with dimensions of 50 mm × 10 mm × 5 mm were created to measure toughness. Five samples from each sample are analysed in each case, and an average is recorded. Following Eq. (1), the stress intensity factor (K_IC_) was computed to represent the fracture toughness^42,43^.

1 $${K}_{IC}=\frac{L}{B{W}^{0.5}}f(x)$$

where

2 $$f\left(x\right)=\frac{6{x}^{0.5}[1.99-x(1-x)(2.15-3.93x-2.7{x}^{2}]}{\left(1+2x\right){(1-x)}^{1.5}}$$

Additionally, B, L, W, and a stand for the respective specimen thickness, load at fracture initiation, specimen width, and crack length. X represents the crack length-to-width ratio.

At temperatures ranging from 30 to 250 °C, using a heat rate of 2 °C per minute at 1 Hz, the mechanical damping factor, storage moduli, and loss moduli of epoxy composites were all examined in tension mode. A DSC Q1000 from TA-Instruments was used to detect the glass transition temperature (Tg) for the dried samples under an environment of N_2_. The samples, which weighed around 6 mg, were heated at a rate of 5 °C/min from 400–250 °C. Using a ramp rate of 5 °C /min in an environment of N_2_, the thermal stability of the samples was tested in a Q-50 thermo-gravimetric analyzer at temperatures between 30 and 800 °C.

All samples were examined in the Antonpar rheometer (MCR 102) for rheological analysis in the parallel plate mode using the PP 25 measuring system (25 mm diameter) with a shear range of 0.01–100 1/s. The strain sweep was completed, and all the outcomes were organized for presentation.

## Results and discussion

### Confirmation of the formation of double hydrophilic graphene nanoplatelets

**Figure 2:**
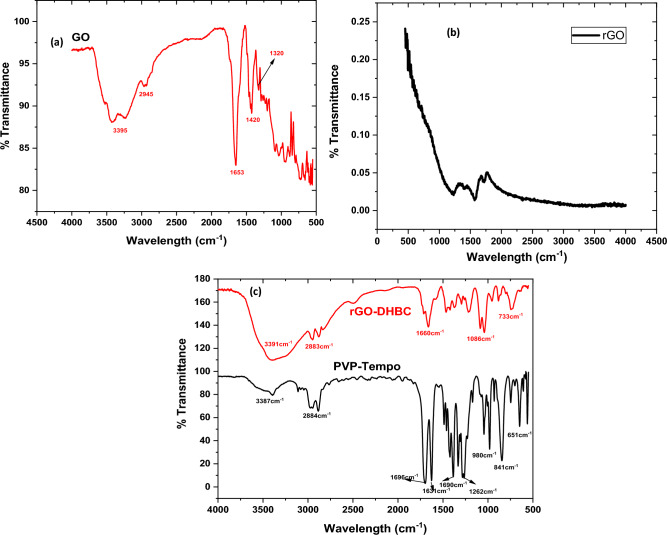
FTIR spectra of (**a**) GO (**b**) rGO (**c**) PVP-TEMPO and rGO-g-DHBC block copolymer.

A thorough FTIR investigation supported the formation of the double hydrophilic nanoplatelets and the reduction of GO. In the FTIR spectra (Fig. 2(a)) of GO, the distinctive band found at 1728 cm^-1^ corresponds to C=O carbonyl stretching, while the peak at 1050 cm^-1^ is caused by C–O stretching vibrations. The oxidation of graphite to graphene oxide is justified by the wide band found at 3400 cm^-1^ that corresponds to OH groups. The adsorbed water may be responsible for the bands at 1621 cm^-1^. The absence of peaks corresponding to carboxylic and hydroxyl groups in the FTIR spectra of rGO (Fig. 2(b)), which are only detected at around 1560 cm^-1^ and 1178 cm^-1^ due to C=C and C–O–C stretching, respectively, confirms the reduction. The skeletal vibrations caused by unoxidized graphitic components can be observed clearly from the FTIR image of rGO. Less intense peaks observed in the case of rGO confirm the reduction process^44^. The C–N stretching is responsible for the peak in rGO-g-DHBC between 3300 and 3500 cm^-1^, and the peak at 1660 cm^-1^ indicates the presence of C=O in the PVP portion of the block (Fig. 2(c)). Similar peaks can be observed in PVP-Tempo, due to the C–N stretching and C=O stretching vibrations. However after the polymerization of PEG at the end of the PVP-Tempo, a broad peak was observed at 3391 cm^-1^ due to the presence of O–H groups in the PEG^26^. The broader peak further confirms the possibility of the formation of intramolecular hydrogen bonding^45^. The C–H stretching vibrations cause the modest peak that was measured at 2800 cm^-1^. The presence of C–H group is confirmed by the peak at 1550 cm^-1^.

**Figure 3:**
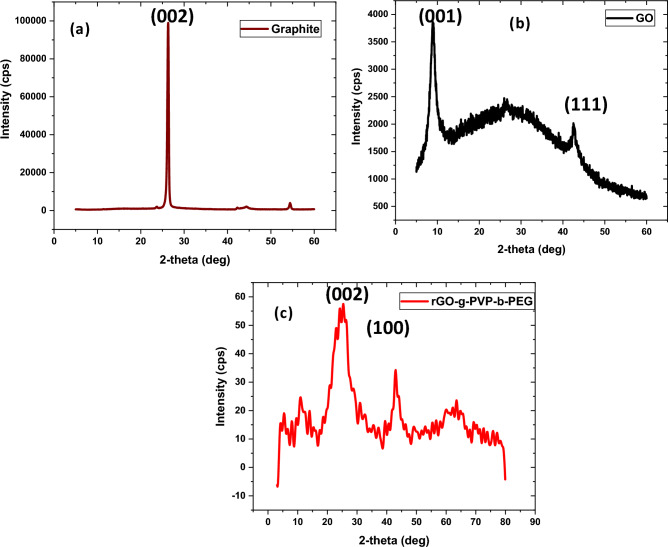
XRD pattern of (**a**) graphite (**b**) GO and (**c**) rGO-g-DHBC block copolymer.

X-ray diffraction (XRD) pattern of graphite shows a peak at 26.315°, which is caused by the (002) plane and corresponds to an interlayer spacing of 0.3384 nm (Fig. 3(a)). The peak is displaced to 9.03° after oxidation using Hummer's approach because of the increase in interlayer spacing (1.27 nm) brought on by the functionalization of the graphite surface as shown in Fig. 3(b). The development of GO is confirmed by the peak at 9.03°. From the absence of the reflection peak at 26.315°, it is confirmed that there are disordered graphitic sheets in the produced GO. The peak for graphite observed at 43° with an interlayer distance of 0.208 nm shows that graphite has no organized structure at all. The peak found at 43° in the instance of rGO-g-DHBC is caused by turbostatic instability in the crystal structure. The reduction of GO to rGO caused the peak at 25° in Fig. 3(c), which is associated with an interlayer distance of roughly 0.37 nm. The pi- pi stacking that occurs between the grafted diblock copolymer chains and graphene nanosheets because of the hybrid formation is confirmed by the increase in d spacing. The pi-pi stacking distance stated in earlier publications is comparable to this d-spacing^30^. The rGO-g-DHBC nano platelets include an amorphous polymer region that causes the peak at 63°. The peak obtained at 13.8°,14.8°, 23.03°, 31.10°, 35.4°, 42.8° etc. are corresponding to the (110), (020), (032), (220), (111) and (200) planes of PEG part which is correlated with JCPDS 04-0783. Peaks obtained at 11° and 20.7° corresponds to the PVP part of the rGO-g-DHBC.

**Figure 4:**
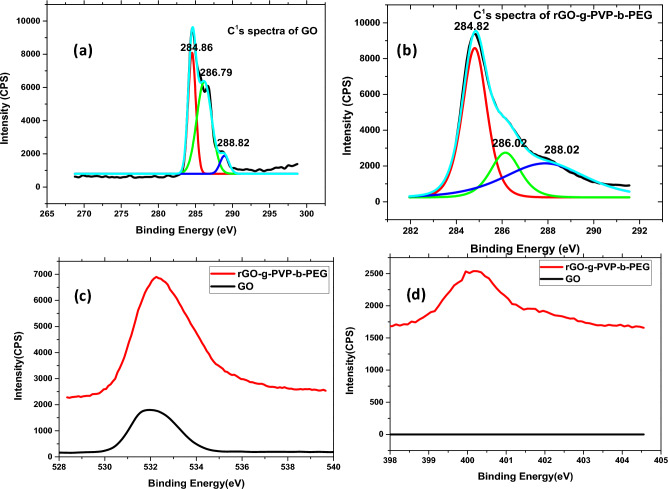
XPS data (**a**) and (**b**) ^1^Cs spectra of GO and rGO-g-DHBC, (**c**) ^1^Os spectra and (**d**) N spectra of GO and rGO-g-DHBC.

The X-ray photoelectron spectroscopy (XPS) gives a clear picture of the formation of double hydrophilic Graphene nanoplatelets. Figure 4(a) and (b) compare the C^1^s spectra of GO with that of the nanoplatelets. In the C^1^s spectra, the peak obtained at 288.82 eV (O–C=O), 286.79 eV (C=O) and that due to the SP^2^ carbon atom (284.46 eV) confirms the formation of GO from graphite (Fig. 4(a)). From the XPS data of rGO-g-DHBC, it is clear that the peak obtained at 288.82 eV is shifted to 288.02 eV in the case of the nanoplatelets and the intensity of the peak is reduced confirming the reduction of GO followed by grafting of the PVP-b-PEG double hydrophilic block copolymer onto it. The intensity corresponding to (O–C=O) and (C=O) are considerably reduced indicating the reduction of GO to rGO. The peak of C=O is shifted to a lower region, due to the grafting of the diblock copolymer which increases the electron density and in turn leads to a reduction in binding energy. The reduced binding energy confirms the grafting process. Even though the GO is reduced, the presence of a long chain of PEG enhances the presence of oxygen. Hence the peak intensity of O^1^s spectra is higher for the rGO-g-DHBC platelets (Fig. 4(c)). Due to the presence of Nitrogen in PVP, the platelets contain a peak in the 400.29 eV, but this peak is absent in GO (Fig. 4(d)). This also helps in the confirmation of the formation of rGO-g-DHBC nanoplatelets.

**Figure 5:**
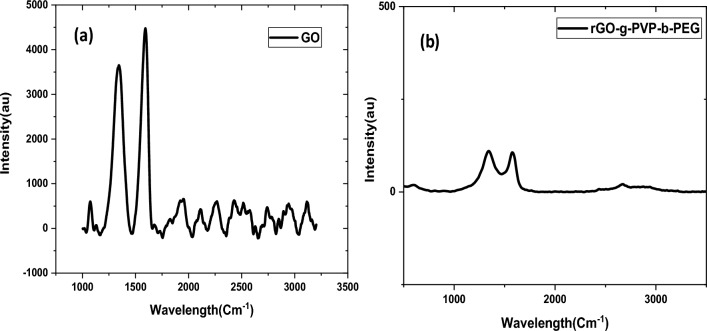
Raman spectra of (**a**) GO and (**b**) GO-g-DHBC block copolymer.

The grafting process is also confirmed by comparing the Raman spectra for GO and rGO-g-DHBC. Chemical modifications can be verified using the intensity of the D and G bands in GO. The change in the intensities of the D and G bands and the change in the intensity ratio are remarkably capable of foretelling the grafting process, as can be observed from the spectra (Fig. 5(a) and (b)). The grafting of block copolymer onto GO is confirmed by a rise in the intensity of the D band and a decrease in the intensity of the G band. Due to the sp^2^ carbon and k-point phonons of the A_1g_ symmetry, the D and G bands have two distinct peaks at 1356 cm^-1^ and 1596 cm^-1^ respectively. The intensity of the D band and G band decreases after the grafting of GO with the block copolymer, which verifies the copolymer's attachment. The increasing I_D_/I_G_ ratio provides more evidence for this. Due to the reduction and subsequent insertion of the copolymer onto GO, the I_D_/I_G_ for rGO-g-DHBC is 1.1 instead of the expected 1 for GO.

**Figure 6:**
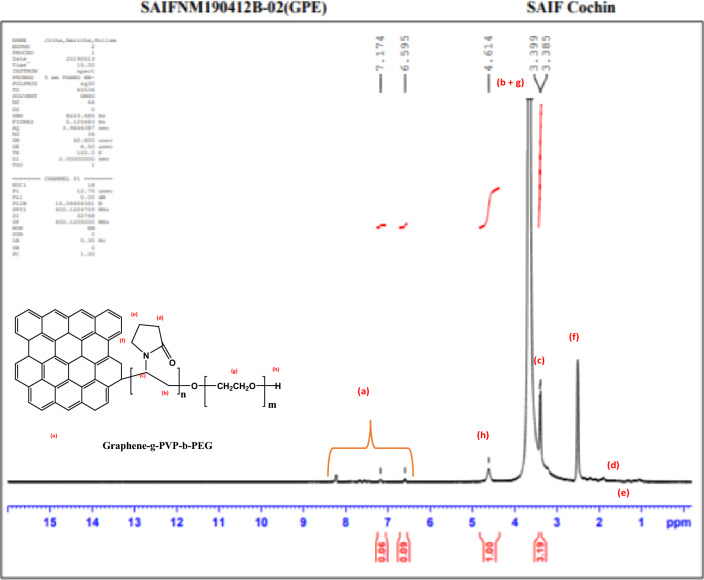
NMR spectra of GO-g-DHBC block copolymer.

Figure 6 displays the nuclear magnetic resonance (NMR) spectra. The peak identified as "a" was obtained at 8.3, 6.5, and 7.1 ppm and is ascribed to the aromatic hydrogen and phenolic groups that are present on the surface of GO. The methine group at the PVP block is seen at a δ value of 3.3 ppm. The methylene groups on the pyrrolidone ring show peaks at δ values 1.3 and 1.6 ppm. The methylene groups present on the PEG block show a peak at 3.5 ppm, which got merged with the ‘b’ proton. The hydroxyl groups at the end of the PEG chain show a peak at δ = 4.5 ppm. Hence the formation of rGO-g-DHBC is confirmed.

### Morphological analysis rGO-g-DHBCs

**Figure 7:**
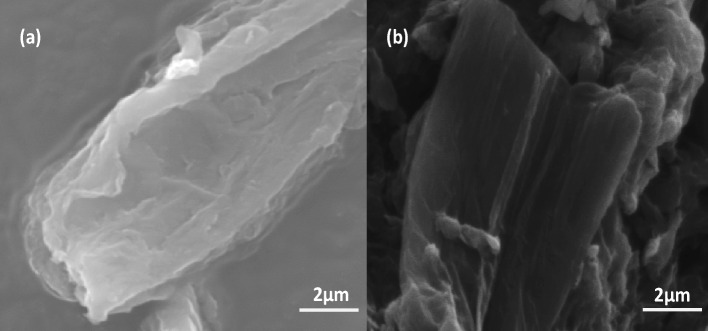
SEM images of (**a**) GO and (**b**) rGO-g-DHBC.

The morphology of the samples was analyzed using various microscopic techniques. From the morphological analysis, it is possible to determine the nature of the arrangement of the graphene sheets in the nanohybrid. Scanning electron microscopy (SEM) images of GO and rGO-g-DHBC upon comparison, show clear change in their morphology. The layered, flake-like, and folded appearance is visible in the SEM image of GO (Fig. 7(a)). After grafting, the flake-like appearance of GO is completely distorted, and the grafting of the block makes the surface amorphous (Fig. 7(b)). The surface distortion, together with the formation of grooves and the fluffy overview enables the confirmation of grafting of the copolymer onto GO. The intercalation of the polymer chains, grafting, and exfoliation of a few GO layers can be clearly understood from the SEM morphology.

**Figure 8:**
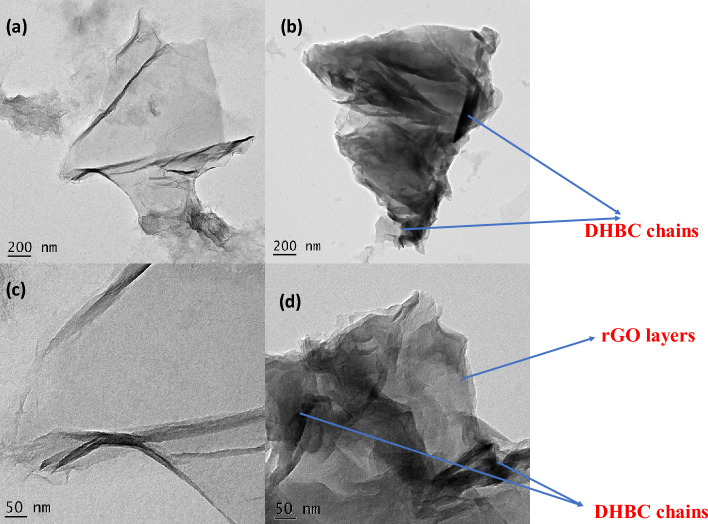
HRTEM images of GO and rGO-g-DHBC (**a**), (**b**) at 200 nm and (**c**), (**d**) at 50 nm resolution.

**Figure 9:**
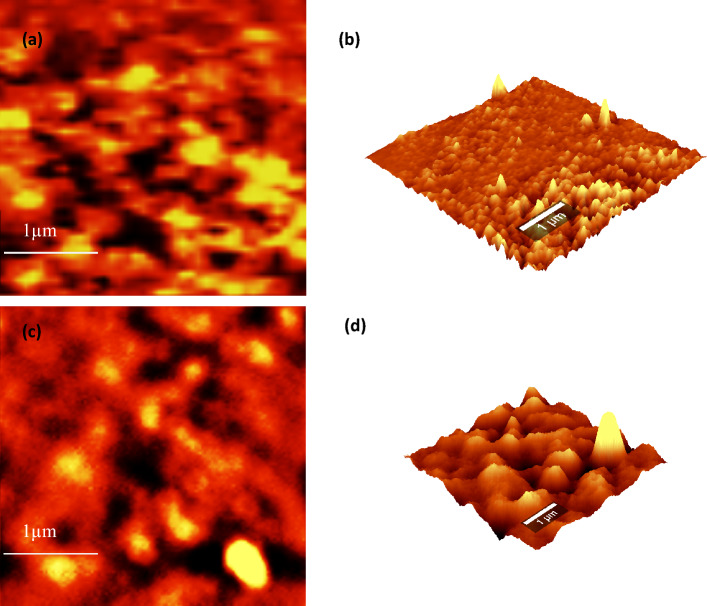
AFM 2D and 3D images of GO (**a**, **b**) and rGO-g-DHBC (**c**, **d**).

High-resolution transmission electron microscopy (HRTEM) images are extremely useful in identifying the grafting process. The GO surface shown in Fig. 8(a) is having a slightly distorted sub-nanometre-sized layered structure whereas the TEM of the grafted filler shows the intercalation of the copolymer within the nanolayers. (Fig. 8(b)). The PVP-b-PEG copolymer has entered into the layers of GO by the process of intercalation (Fig. 8(c) and (d)). The layer-by-layer appearance of GO and the insertion of copolymers into it is observed in the figure. This substantiates the earlier result of the increase of d spacing in the XRD of the rGO-g-DHBC from that of GO. The findings from XRD and TEM are additionally supported by Atomic Force Microscopic (AFM) images. The surface of rGO-g-DHBC had more protrusions than GO, indicating that the copolymer was grafted onto it. Figure 9 displays 2D (Fig. 9a and c) and 3D AFM images of GO and rGO-g-DHBCs. The enhanced skewness of the surface after grafting the polymer into the chain counts the roughness of the sample. The SSK value for GO was obtained as 1.4, but after grafting the chains of PVP-b-PEG copolymer, it was further changed to 2.8, confirming the protrusions after the polymer grafting; this is further evident from Fig. 9(b) and (d). However, after the incorporation of DHBC, the lamellar or the layered structures are lost due to the insertion of the polymers into the layers of GO, indicating exfoliation after the grafting process.

### Mechanical properties of epoxy composites

#### Fracture toughness

**Figure 10:**
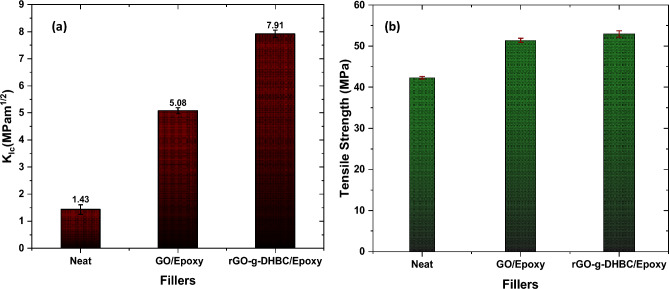
(**a**) fracture toughness and (**b**) Tensile strength of neat, GO and rGO-g-DHBC epoxy nanocomposites.

To examine the effect of GO and double hydrophilic block copolymer grafted GO in enhancing the mechanical properties of epoxy, GO/epoxy nanocomposite and rGO-g-DHBC epoxy nanocomposites were made and the mechanical properties were analyzed. It is understood from our previous studies that 0.1 wt% loading of GO can show better properties compared to other loadings (Table S1 in supplementary information); Therefore, the same loading is used for comparing the mechanical properties. The observed results are exciting and are shown in Fig. 10(a). From the figure, it is clear that the composites containing the grafted filler exhibit superior properties than the neat and GO-loaded epoxy composites. Neat epoxy shows a stress intensity factor of 1.42 ± 0.07 MPam^1/2^, but as GO is loaded, the toughness shows a hike to 5.56 ± 0.2 i.e. 290% improvement is obtained. Interestingly upon the addition of rGO-g-DHBC, an improvement of about 457% was observed when compared to that of neat epoxy. The stress intensity factor obtained was 7.91 ± 0.48. The load and the K_IC_ values are tabulated in S2 (Table S2 in supplementary information).

The effect of fillers on the tensile properties was also monitored to check whether the addition of the DHBC affects the inherent properties of the epoxy. The tensile results (Fig. 10(b)) show that the incorporation of rGO-g-DHBC has not reduced the tensile properties of epoxy. 21.51% improvement was observed for the GO-added epoxy, while in the case of rGO-g-DHBC toughened epoxy, the tensile strength showed an improvement of about 25.06% than the neat epoxy. The load vs displacement graphs are provided in the supplementary file (Figure S1 in supplementary information).

These results (Table 1) highlight the synergistic effect of GO and the DHBC in enhancing the toughness and mechanical strength of the epoxy resin. This can be attributed to the better miscibility of GO after the grafting process. Moreover as reported, PEG and PVP are miscible with the epoxy matrix individually^51,52^. The individual blocks are capable of enhancing the toughness of epoxy independently. Moreover, the GO, upon association with the DHBCs to form the nanoplatelets exhibits an intercalated morphology and gets aligned more easily in the stress direction of the epoxy matrix leading to an enhancement in toughness as well as tensile strength.

**Table 1:** Fracture toughness and tensile strength in comparison with existing polymer/GO toughened epoxy systems.

Name of filler	Loadings	% improvement in toughness	Tensile strength	References
GO	0.1	255	21% improvement	Previous work^46^
PVP	0.1	148	12% reduction	Previous work^33^
PEG	1	213	8.7% improvement	Previous work^31,32,34^
GO-g-PEG	0.1	334	3.3% improvement for 0.1 wt% and 12% for 0.5 wt%	Previous work ^46^
GO-g-PVP	0.1	190	12% improvement	Previous work^33^
GO-g-PAA	0.7	87	–	Sahu et al. ^47^
GO-g-CTBN	0.6	128	25% Improvement	Konnola et al. ^48^
GO-g-DGEBA	0.25	26	75% Improvement	Wan et al.^49^
GO/PEO-PPO	0.04 Wt% of GO + 5 wt% PEO-PPO	170	0% Improvement	Li et al. ^16^
rGO/PCL-PPC-PCL	0.04 Wt% of GO + 30 wt% PPL-PPC-PPL	60%	55% Reduction	Liu et al.^50^
rGO-g-DHBC	0.1 wt%	457%	25% Improvement	Current work

#### Toughening mechanism

**Figure 11:**
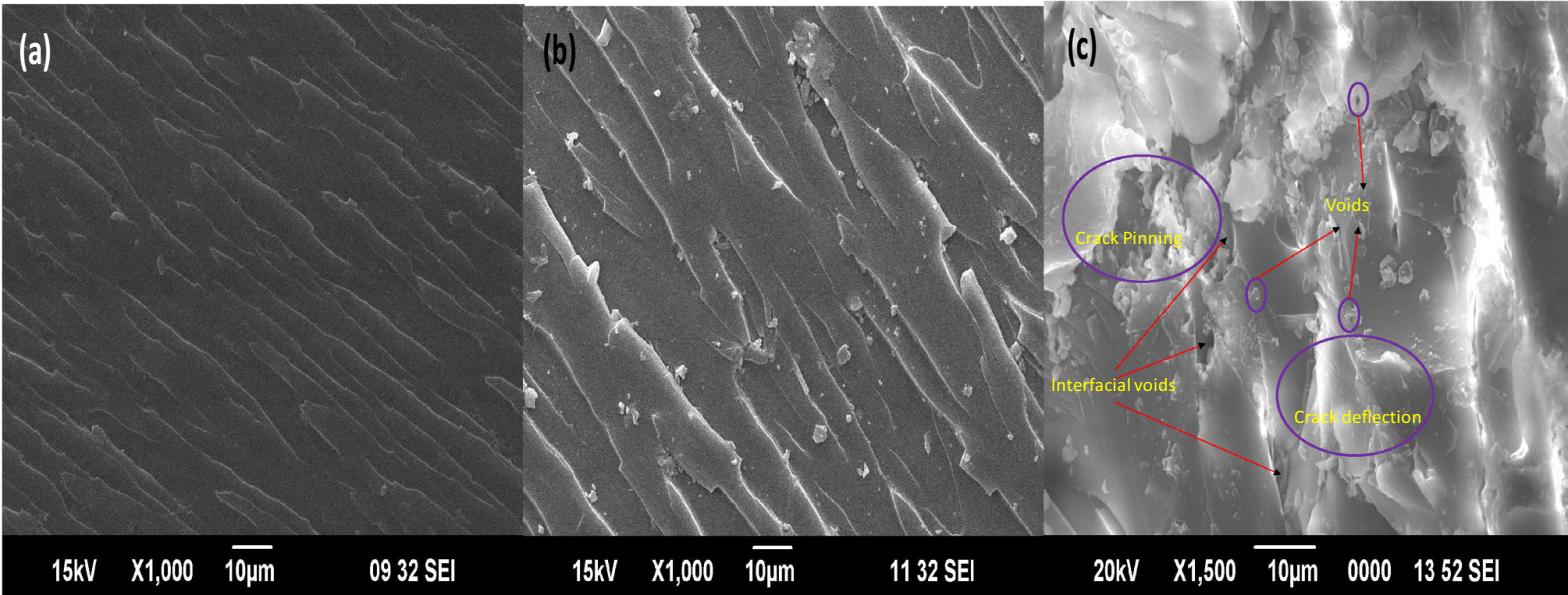
SEM images of fractured surface (**a**) neat, (**b**) GO and (**c**) rGO-g-TBCP toughened epoxy composites.

The toughening mechanism is explained in detail with the help of SEM images of the fractured surface. From the fractographs, it is clear that the neat epoxy (Fig. 11(a)) is prone to brittle fracture due to the plane-fractured surface^4^. This supports the observation of poor fracture toughness of the neat system, whereas in the GO-loaded epoxy, the toughness is enhanced, and this is attributed to the crack pinning and crack deflection as observed in the SEM images (Fig. 11(b)). The presence of sub-cracks and microcracks in the GO toughened epoxy system enhances the toughness to a great extent^34^. The presence of coarseness and ditches in the SEM image confirms the crack deflection where the cracks twisted and tilted from the direction of propagation. But in the case of the rGO-g-DHBC toughened epoxy system, the fractured surface shows the development of voids beyond the crack deflection and crack pinning (Fig. 11(c)). The presence of voids by the debonding of the particle from the surface of epoxy helps in the drastic enhancement of toughness due to the presence of rGO-g-DHBC in epoxy. Hence the grafting of the double hydrophilic polymer enhances the toughness by improving the debonding and the particles get pulled out from the surface. More energy is required for the pull-out process and hence the stress intensity shows an increase.

**Figure 12:**
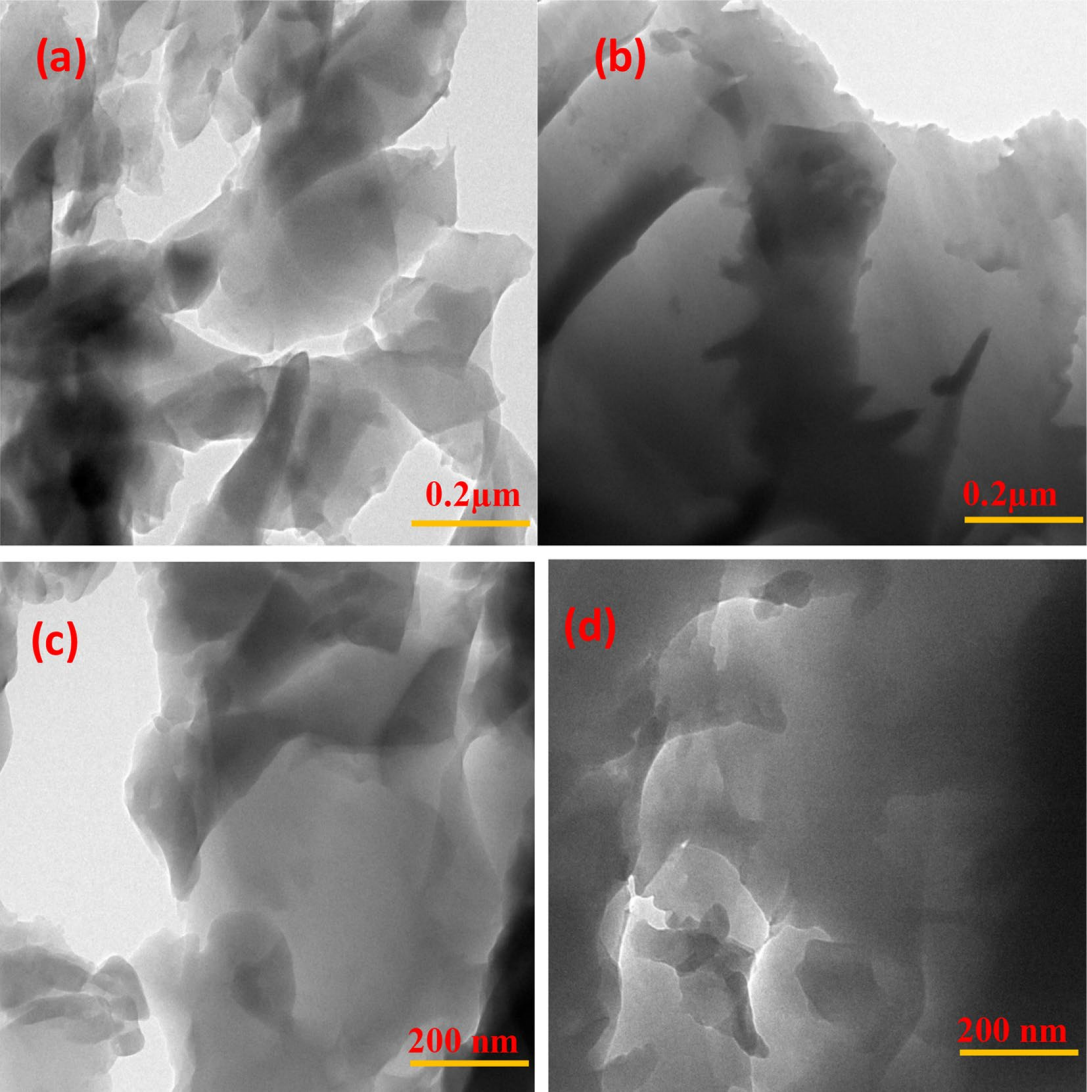
HRTEM images of ultramicrotome cross-section of neat at (**a**) 0.2 µm (**c**) 200 nm and of rGO-g-DHBC epoxy composite at (**b**) 0.2 µm and (**d**) 200 nm.

HRTEM images of the ultra-microtomed cross-section of the fracture surface were taken and these images (Fig. 12) helped in revealing the behaviour of rGO-g-DHBC in the epoxy matrix. As mentioned before, the rGO-g-DHBC mixes well in the epoxy matrix due to the double exophilic nature of the double hydrophilic PVP-b-PEG block copolymer. Hence the grafting of the block copolymer to GO, helps it to disperse well in the epoxy matrix^53,54^. The HRTEM images of neat and rGO-g-DHBC toughened epoxy system show a similar appearance indicating better dispersion of the nanoplatelets in the epoxy matrix as shown in Figure S2. Owing to the epoxiphilicity of both ends rather than forming micelle as in triblock copolymer grafted GO^8^, double hydrophilic graphene nanoplatelets show better interaction with the epoxy and get dispersed well. Thus, the well-dispersed rGO-g-DHBC is capable of enhancing the toughness of epoxy without compromising the tensile properties.

#### Dynamic mechanical analysis (DMA)

**Figure 13:**
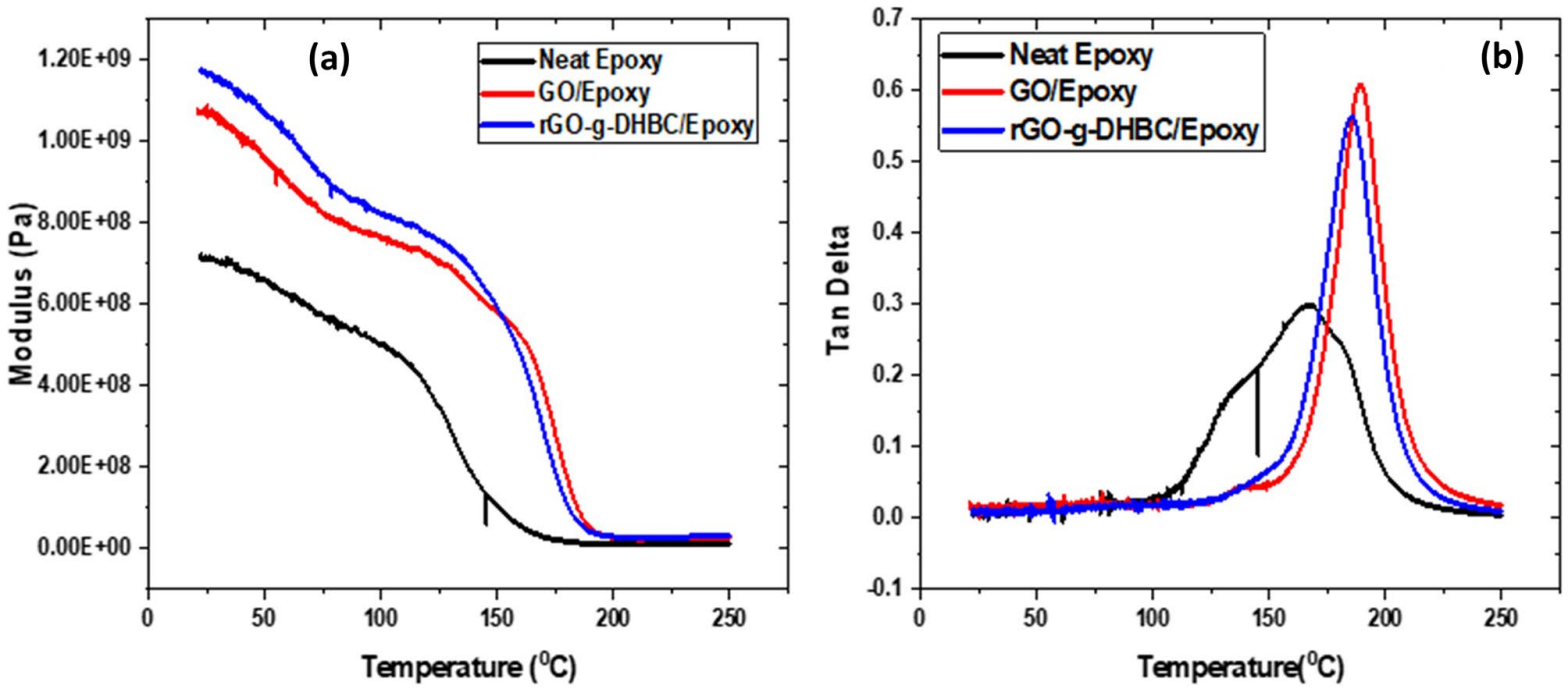
(**a**) Storage modulus and (**b**) Tan δ curve of neat epoxy, GO/epoxy and rGO-g-DHBC/epoxy composites.

The DMA analysis shows that the storage modulus of the epoxy composites increases with the addition of GO. The increase in storage modulus is because of the interaction of GO and epoxy (Fig. 13(a)). Thus, the filler restricts the motion of individual polymer chains. The modification of GO using the DHBC further enhances the storage modulus. Generally, it is observed that the block copolymer toughened epoxy reduces the modulus^55,56^. The grafting of DHBC enhances the storage modulus to 70% compared to neat epoxy, but GO shows an enhancement of only 51%. The block copolymer content is capable of enhancing the stiffness of epoxy by increasing the modulus of GO. On comparing the tan δ curves, (Fig. 13(b)) it can be confirmed that the energy dissipation of epoxy increases with the addition of GO as well as DHBC-modified GO. However, the graft reduces the confinement of polymer chains by enhancing the chain mobility. This is because of the reduction followed by the intercalation of DHBC to the GO layers. Hence the intensity of the tan δ peak for GO toughened system gets reduced. The Tg value calculated from the tan δ curve confirms the same. The Tg value got reduced to 185 °C from 195 °C of GO toughened epoxy, due to the enhanced chain mobility. The neat epoxy shows a Tg of about 165 °C and hence it is clear that the two fillers are capable of enhancing the glass transition.

The crosslink density of the composites is calculated by the equation mentioned in our previous study^8^, and compared in Table S3, it is clear that the improvement in Tg is attributed to the improvement in the molecular weight between the crosslinks. The strong interaction of the filler with the matrix attributes enhanced interfacial interaction between the fillers and the matrix, thus Tg will get improved. The incorporation of GO reduces the crosslink density but due to the inherent mechanical strength and compatibility of GO in the epoxy matrix; it can show enhanced mechanical properties and stiffness. The grafted system shows an increased crosslink density; thus, the stiffness is improved. The synergistic effect of DHBC and graphene further helps in improving the mechanical properties. The improved cross-link density of rGO-g-DHBC in epoxy ensures better compatibility of the system with epoxy^57,58^. The lower crosslink density of rGO-g-DHBC/epoxy system over the other two systems is the reason behind the improved toughness. In the case of DHBC, the crosslink density is lower which suggests better toughenability. As higher the molecular weight between the crosslinks (Mc) value, the higher the K_IC_. Similar to the case of BCPs toughened epoxy, rGO-g-DHBC toughened epoxies show the same trend, lower crosslink density promotes the toughenability^59^.

### Thermal properties

**Figure 14:**
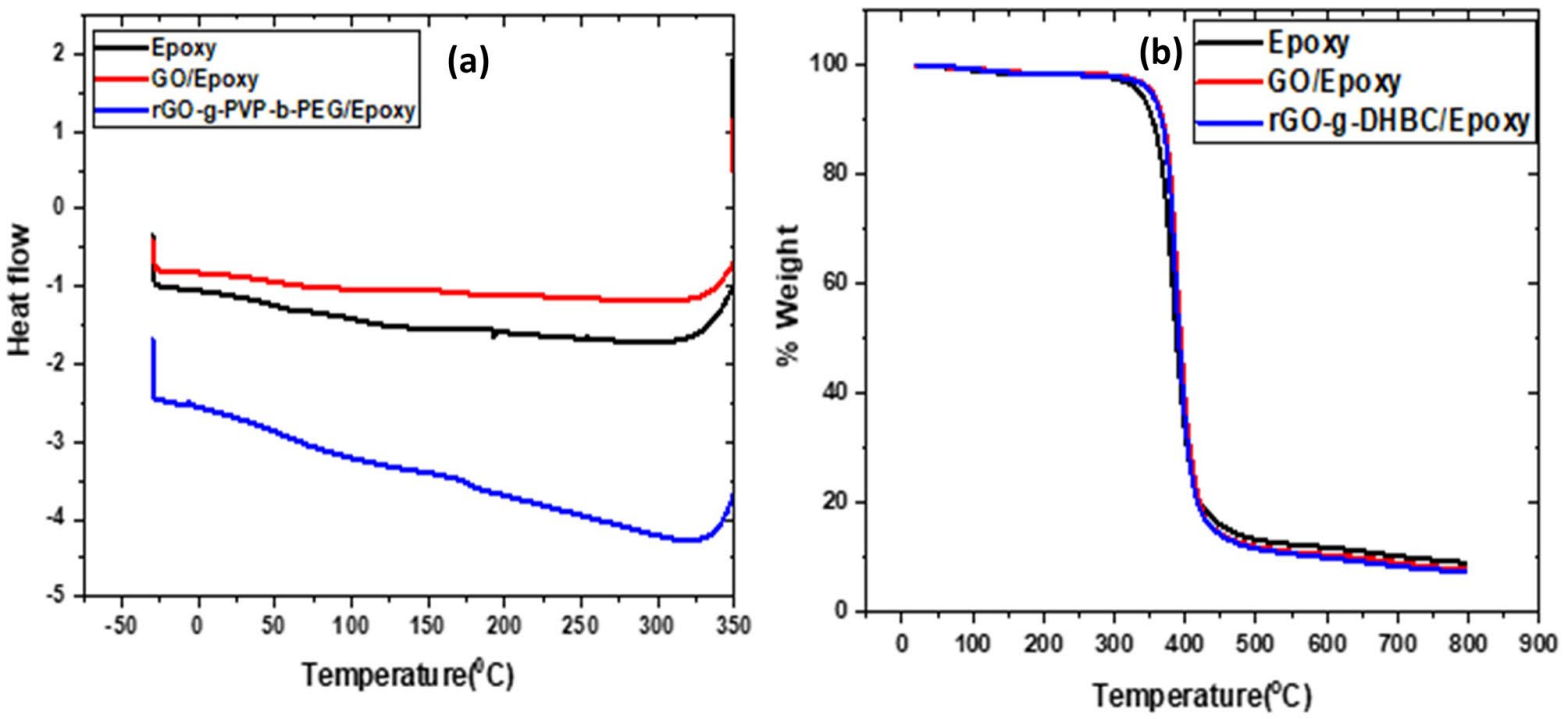
DSC and TGA curve of neat epoxy, GO/epoxy and rGO-g-DHBC/epoxy composites.

Figure 14(a) shows the Differential Scanning Calorimetry (DSC) curves obtained for neat epoxy, GO and rGO-g-DHBC epoxy composites. The addition of these nanofillers is capable of enhancing the glass transition temperature due to the better interaction of filler with the matrix. The increase in Tg is associated with better interaction of the filler with the matrix. In case of neat epoxy, the Tg is only 95 °C , but with the incorporation of GO and rGO-g-DHBC, the Tg value changes to 165 °C and 150 °C respectively. The presence of functionalities over GO ensures better interaction with epoxy. This further immobilizes the epoxy chain and thus produces an enhancement in toughness^60–62^. But the grafting of DHBC after the reduction of GO reduces its interaction with epoxy. The presence of reactive functionalities on DHBC reduces the crosslink density and hence the reduction in Tg is not a pronounced one.

The thermal stability of rgO-g-DHBC/epoxy composites was determined with the help of Thermogravimetric Analysis (TGA) and the results are compared with that of GO/epoxy composites. The results show (Fig. 14(b)) that the incorporation of the nanofiller is capable of maintaining the thermal stability of epoxy composites. Nevertheless, the addition does not bring a tremendous improvement in stability. The neat epoxy starts to degrade at a temperature of 320 °C but the incorporation of GO as well as rGO-g-DHBC enhances the stability to about 370 °C i.e. the degradation temperature is slightly improved.

### Rheology

**Figure 15:**
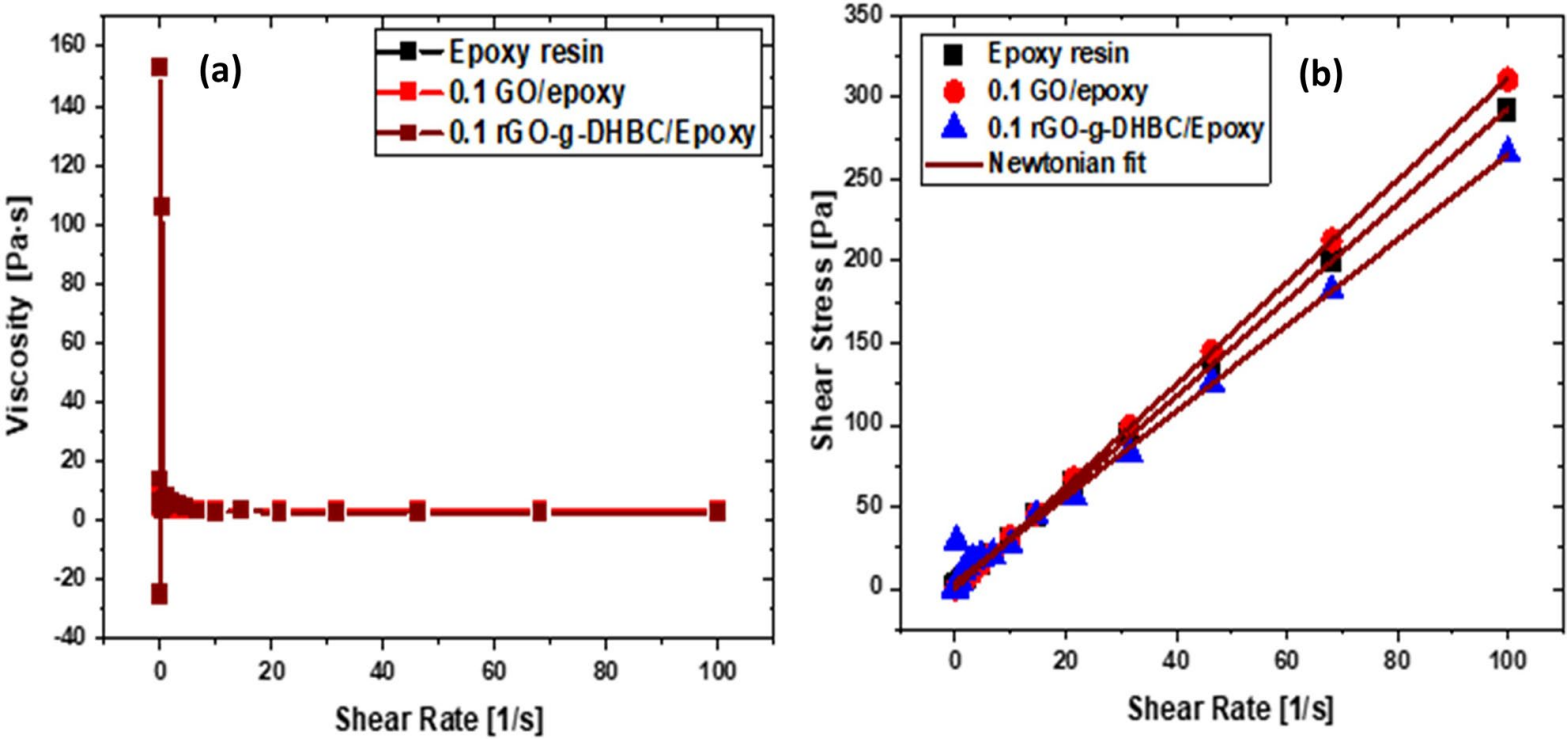
(**a**) Viscosity of epoxy, GO/epoxy and rGO-g-DHBC/epoxy as a function of shear rate.

The fillers are incorporated into the uncured epoxy resin and the flow characteristics of the uncured samples are analyzed in detail. Rheological analysis shows that the incorporation of filler does not alter the viscosity of epoxy resin (Fig. 15(a)). This shows that the GO and the DHBC grafted GO can disperse well in the epoxy matrix and thus the flow behavior of epoxy is maintained. Further, the flow behavior of epoxy is modeled using the Newtonian model^32^. The fitting curves are shown in Fig. 15(b), and the curves indicate that the incorporation of GO as well as rGO-g-DHBC does not alter the Newtonian behavior of epoxy. i.e. the shear rate does not have any effect on the viscosity of epoxy. The fillers are not causing any change in the Newtonian behavior of epoxy as well.

## Conclusion

In conclusion, double hydrophilic-based graphene nanoplatelets were successfully synthesized by nitroxide-mediated polymerization. The synthesis of the resultant materials was confirmed by the help of FTIR, XRD, Raman, NMR, and XPS spectra and presented in detail. The reduced graphene oxide grafted hydrophilic block copolymers were subsequently utilized as toughening agents in the epoxy matrix. The double hydrophilic nano platelets exhibited an improvement of ~ 457% in fracture toughness and ~ 25.05% in tensile strength. The non-covalent interaction leading to pi-pi stacking of the nanographene platelets together with the intercalation of the diblock copolymer within the graphene platelets account for the increased mechanical performance exhibited by the system. The miscibility of the blocks in the epoxy also contributes positively towards the improvement of mechanical properties. These superior properties will surely enhance the utility of epoxy nanocomposites enabling them as a better choice for high-end applications involving aerospace and defence. The Tg value of the system was improved by the addition of the filler which is confirmed with the help of DMA and DSC. From the DMA analysis, it is confirmed that the incorporation of DHBC does not affect the storage modulus of the epoxy system. The study also correlates the crosslink density and toughness of epoxy systems, the rheological analysis further proves the Newtonian behavior of the system. This research will open new avenues toward the fabrication of tailored hybrids with unique features for the strengthening of epoxy resins.

### Supplementary Information

Supplementary Information.

## Data Availability

Data will be available on request to authors.
