# Supplementary material for: Designed and tailor-made double hydrophilic block copolymer-graphene nanoplatelet hybrids for reinforcing epoxy thermosets
Source: Sci Rep. 2024 Apr 16;14:8812. doi: 10.1038/s41598-024-59322-x (PMC11021408; doi:10.1038/s41598-024-59322-x)
Supplement: Supplementary file 1 — Supplementary Information. [file 41598_2024_59322_MOESM1_ESM.docx]

**Designed and Tailor-made Double Hydrophilic Block Copolymer-Graphene Nanoplatelet Hybrids for Reinforcing Epoxy Thermosets**

Jitha S Jayan^1^*, BDS Deeraj^2^, Kuruvilla Joseph^2^*, Appukuttan Saritha^1^*

*^1^Department of Chemistry, Amrita Vishwa Vidyapeetham, Amrita University, Kollam, Kerala, India*

*^2^ Department of Chemistry, Indian Institute of Space Science and Technology Valiyamala, Thiruvananthapuram, Kerala, India*

*jithasjayan7652@gmail.com, sarithatvla@gmail.com, kjoseph.iist@gmail.com*

***Contents***

Table S1 – Page S2

Table S2 – Page S2

Table S3 – Page S3

Figure S1– Page S3

Figure S2 – Page S4

***Table S1:*** *Comparison of Fracture Toughness and Tensile Strength of different weight percentages of GO-loaded Epoxy Systems*

| **Filler loading of GO (wt%)** | **Fracture Toughness (K_IC_)(MPam^1/2^)** | **Tensile Strength (MPa)** |
| --- | --- | --- |
| **0** | **1.43±0.2** | **42.3±0.1** |
| **0.05** | **3.90±0.2** | **49.3±0.1** |
| **0.1** | **5.08±0.11** | **51.4±0.1** |
| **0.3** | **4.53±0.1** | **46.3±0.1** |
| **0.5** | **2.72±0.1** | **52.86±0.1** |

***Table S2:*** *The* *calculated fracture toughness in terms of stress intensity factor (K_IC_) of epoxy systems*

| Load(N) | K_IC_(MPam^1/2^) |
| --- | --- |
| Neat Epoxy | |
| 41.39 | 1.31 |
| 50.51 | 1.61 |
| 50.67 | 1.60 |
| GO/Epoxy | |
| 164.31 | 5.2 |
| 153.82 | 4.9 |
| 164.33 | 5.2 |
| rGO-g-DHBC/Epoxy | |
| 280.63 | 8.87 |
| 233.74 | 7.39 |
| 236.16 | 7.47 |

***Table S3:*** *Crosslink density of epoxy nanocomposites calculated from DMA.*

| System | Loading(wt%) | Tg(ºC) | Crosslink density(ⱱ) (mol/m^3^) | Molecular weight between the crosslinks (Mc) |
| --- | --- | --- | --- | --- |
| Neat epoxy | 0 | 165 | 3820 | 0.0001 |
| GO/epoxy | 1 | 185 | 1982 | 0.0002 |
| rGO-g-DHBC/epoxy | 1 | 195 | 1806 | 0.001 |

***Figure S1****: Load vs displacement graph of epoxy nanocomposites*

*Figure S2: Schematic representation of the arrangement of nanoplatelets in epoxy matrix*
